# Hip fracture care and national systems: The United States and Canada

**DOI:** 10.1097/OI9.0000000000000073

**Published:** 2020-03-23

**Authors:** Todd Swenning, Jennifer Leighton, Michelle Nentwig, Bradley Dart

**Affiliations:** a Desert Regional Medical Center, Palm Springs, CA; b 304-1540 Summer St., Halifax, NS; c Department of Orthopedics, University of Kansas-School of Medicine, Wichita, KS.

**Keywords:** geriatric fractures, hip fractures, hip fracture systems

## Abstract

While it is widely understood that management of hip fractures not only represents clinical decision making dilemmas for the individual orthopaedist, these increasingly common injuries present economic burdens to local and national systems as well. This supplement article looks at current clinical trends, as well as systems-based issues in the United States and Canada.

## 1 Introduction

It is widely accepted that geriatric hip fractures are at endemic levels. While hip fractures occur more commonly in Europe, Canada, and the United States, it is expected that these numbers will increase globally due to demographic changes.^[2]^ Annually, it is estimated that there are between 260,000 and 300,000 admissions for hip fractures in the US, with projections of more than 500,000 per year by 2040.^[3,5,16]^ While Canada has a smaller population, the incidence and projections follow similar trends.^[4]^ While the United States and Canada have certain distinctly different methodologies in health care delivery, the principle tenets of hip fracture management are quite similar.^[15]^

## 2 Clinical considerations

### 2.1 Initial preoperative assessment

Upon initial consultation for hip fracture, a thorough preoperative assessment is undertaken, as per any surgical patient. Specific to the hip fracture patient, however, it has been recognized that additional assessment for falls risk, pressure ulcer risk (particularly if the patient is coming from the nursing home setting), and screening for delirium, dementia and depression, are all of particular importance given their effects on the recovery trajectory of the hip fracture patient. Documentation of the patient's goals of care is performed for each patient—ideally at the time of admission. Basic screening lab work and appropriate radiographs are undertaken, but additional tests in this patient population may include nutritional markers, markers for bone health, chest radiographs, and/or Electrocardiogram. Consultation of appropriate services can then be undertaken based on test results.^[18]^

### 2.2 Timing

The controversy surrounding surgical delays focused historically on data suggesting increased time to surgery was associated with increased morbidity and mortality. While true, many of the early studies did not account for the difference between delays due to surgeon/OR availability and true medical delays. More recent data suggest risk stratification to guide surgical timing.^[11]^ While the American Academy of Orthopedic Surgeons Guidelines for Hip Fracture Management gave a moderate recommendation for surgical treatment within 48 hours, many institutions strive to achieve timing goals closer to 12 to 24 hours postinjury.

In Canadian centers, guidelines are provincially based, but all currently aim for time to surgery of under 48 hours. This is based upon the 2010 guidelines from the Canadian Bone and Joint Health Network.^[18]^ These guidelines include perioperative checklists to optimize patient care in the pre-, intra-, and postoperative periods, and highlight the negative impact of delay to surgery beyond 48 hours. As further evidence emerges citing increasing complications with wait times over 24 hours, there is a movement amongst orthopaedic surgeons to push for tighter targets.^[13]^ Currently, one-third of patients are receiving surgery within 24 hours of presentation, with specific barriers including the need for hospital transfer and the request for a preoperative echocardiogram.^[13]^ Room for improvement is ample, with multiple national and provincial studies ongoing in this area.

### 2.3 Anticoagulation management/management of anemia

Anticoagulants and the role of reversal therapy are a frequently debated topic by those involved on the care team. It has been thought that operating on patients with an INR >1.5 would lead to an increase in transfusion rates and postoperative complications. While there is no standard for ideal INR that is safe for surgery, recent trends and indeed literature suggest that operating through INR elevated as high as 2–3 has no significant effect on blood loss or the need for transfusion.^[7]^ Options for reversal of INR include IV or oral vitamin K, Fresh Frozen Plasma, or Prothrombin Complex Concentrate. Although previous studies have shown an increase in time to surgery for patients taking Coumadin, early initiation of reversal therapy tends to normalize these delays.

There is limited evidence to support the delay of surgery for patients taking aspirin or antiplatelet medications; however, most of these are withheld the day of surgery and often resumed postop day 1 or 2. The most effective strategy to manage the anticoagulated patient is to build specific algorithms into the preoperative assessment allowing hard stops for when surgery should be delayed. These algorithms may be hospital-based, or state-/province-based.

Often, these patients suffer from chronic anemia. Current recommendations include avoiding transfusion, unless the patient's hemoglobin falls below 8 g/dL. Clearly, this is a case-by-case basis and other risk factors and comorbidities need to be considered.^[16]^

### 2.4 Anesthesia/pain control

Geriatric patients are generally opioid-sensitive and minimization of narcotic utilization is encouraged. Methods such as pre- and postoperative regional anesthesia, multimodal nonnarcotic medications (both pre- and postoperatively), cognitive measures, breathing techniques, and use of ice should all be maximized. Alternative therapies such as music therapy are also garnering a small, but increasing, base of support. General vs spinal anesthetic techniques should be selected based on individual circumstances. While there is conflicting evidence to support one over the other, if general anesthesia is chosen, judicious use of narcotics is warranted. In the area of postoperative delirium, best evidence would suggest that regional anesthetic techniques result in decreased postoperative confusion.^[12,18]^

### 2.5 Surgical options

Surgical options for treating geriatric hip fractures vary widely. Fracture type, patient factors, and surgeon experience all play a role in the decision as to how to best manage hip fractures. While minimally displaced injuries can be managed with percutaneous screw fixation, elderly patients with displaced femoral neck fractures should be managed with arthroplasty.

Debate exists regarding unipolar vs bipolar vs total hip arthroplasty as to best practice. While unipolar and bipolar arthroplasty appear to have similar outcomes and complication rates, total hip arthroplasty may provide better functional outcomes. Surgeon preference and patient factors such as activity level, bone quality, and medical comorbidities must all be considered in elder femoral neck fracture management.^[16]^

Fractures involving the peritrochanteric region of the femur are typically reduced and stabilized with either intramedullary fixation or sliding hip screw fixation depending on surgeon preference and fracture stability. In more stable peritrochanteric fractures, surgeon preference and to a lesser extent cost considerations seem to be dictating the choice between sliding hip screws and cephalomedullary nails. While sliding hip screw constructs are less expensive, there are definite trends toward nailing of these fractures.^[1]^ Management of unstable fractures and reverse oblique type patterns should be and generally are managed with cephalomedullary devices.^[16]^

### 2.6 Postoperative management

Postoperative goals aim to initiate early mobilization and immediate weight bearing. This task, often accomplished as early as a few hours after surgery, has been shown to decrease the risk of postoperative complications such as delirium, infections (pneumonia and urinary tract infections), and deep vein thrombosis. Early mobilization also facilitates early discharge planning by providing an assessment of potential postoperative needs. Programs that streamline this process and include a multidisciplinary team of therapists and social workers have shown improved outcomes and decreased the length of stay.^[8,14]^

In order to achieve these benefits, immediate weight bearing is initiated, and has been demonstrated to be safe, with modern implants and techniques. Notably, patients have been demonstrated to self-limit their amount of weight bearing in more unstable fracture patterns, and thus full weight-bearing status is appropriate for all hip fracture patients.^[10]^

## 3 Systems considerations

### 3.1 Geriatric hip fracture programs/orthogeriatric comanagement

Geriatric hip fracture programs and Orthogeriatric comanagement systems are increasing in number. While the general concepts of interdisciplinary care, clinical protocols and pathways, osteoporosis screening, and education are all understood, there is currently no standardized definition of these programs.

The 3 models that are most widely used include: Shared Care—comanagement by both orthopaedic and geriatric teams; Orthopaedic care with geriatric consultation; Geriatric care with orthopaedic consultation. Each of these models has demonstrated improved patient outcomes and patient mortality rates, though the optimal model for care remains to be determined.^[8]^ Despite the uncertainty regarding the optimal model for comanagement, it remains clear that dedicated Geriatric Fracture Programs have been shown to have positive effects on time to surgery, patient mobility, prevention of adverse events, length of stay, and in-hospital mortality.^[8,9,14]^ More studies are needed, but it appears that correlations with reduced costs and decreased readmission rates are present in efficiently managed Geriatric Fracture Programs as well.^[9]^

These benefits have been demonstrated in the Canadian, American, and European literature.^[8,9,14]^ Ongoing research to determine optimal models of care in each setting is ongoing. Currently, these models are determined on a regional basis, and are affected most by funding models, while in Canada, physician work force availability is an additional factor.

###  2.2 Osteoporosis diagnosis and treatment/fracture liaison service

Screening for osteoporosis and implementation of treatment should be considered standard of care in geriatric fracture patients. Fracture liaison service implementation is used to ensure appropriate osteoporosis screening, fall prevention education, home safety evaluation, nutritional guidance, vision assessment, and social support. Most commonly, this involves the use of specially trained nurses to evaluate patient risk and implement an osteoporosis treatment pathway.^[6]^ This has been shown to significantly improve rates of diagnosis and treatment of osteoporosis, compared with conventional models.^[17]^ This, in turn, decreases the rates of recurrent fragility fracture, and has demonstrated cost effectiveness in the Canadian single-payer health care model.^[19]^ American data on cost-effectiveness has shown early promise, but more rigorous evaluation is ongoing.^[9]^

### 2.3 Registries/quality improvement initiatives

While there is no primary nationalized registry in either the United States or Canada, both countries do have various registries and quality improvement programs in which surgeons may participate. Bone and Joint Canada has a registry and toolkits for starting Geriatric Fracture Programs. Arthroplasty patients—including both hemiarthroplasty and total hip arthroplasty for fracture, are captured by the Canadian Joint Replacement Registry, in association with the Canadian Institute for Health Information. The principle tracking options in the US are Own the Bone (American Orthopaedic Association), Orthopaedic Trauma Association trauma registry, National Surgical Quality Improvement Project (NSQIP), and Trauma Quality Improvement Project (TQIP). NSQIP and TQIP are both run under the auspices of the American College of Surgeons. A recent look at the data from nonspecific registries has demonstrated the need for a more disease-specific collection methodology in order to better comprehend the clinical and financial aspects of hip fracture care.^[5]^

### 2.4 Summary

The role of the orthopaedic surgeon should not stop after surgical repair is complete. We have a unique opportunity to impact many other areas in this fragile population. Osteoporosis, vision, balance, and many other diagnoses critical to a patient's fall/fracture risk are often most successfully addressed while in the Acute Hospital setting. The acute setting may not be the most appropriate setting for definitive intervention but will afford the most opportune time to initiate changes and offer education. While the most well-planned protocol for treating a patient with a fractured hip may result in a complete recovery, the fracture that was prevented affords the best outcome.
